# *QuickStats:* Percentage* of All Emergency Department (ED) Visits^†^ Made by Patients with Asthma,^§^ by Sex and Age Group — National Hospital Ambulatory Medical Care Survey, United States 2014–2015

**DOI:** 10.15585/mmwr.mm6705a5

**Published:** 2018-02-09

**Figure:**
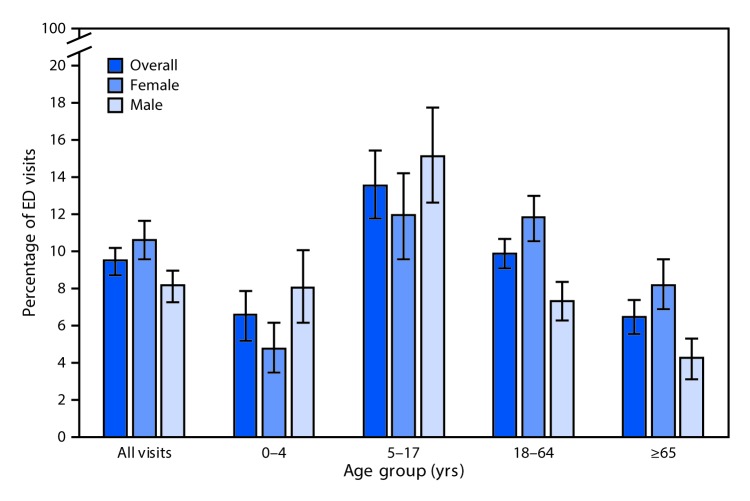
During 2014–2015, patients who had asthma documented in the medical record accounted for 9.5% of all ED visits in the United States, with the highest percentage for children aged 5–17 years (13.6%), compared with 6.6% for children aged 0–4 years, 9.9% for adults aged 18–64 years, and 6.5% for those aged ≥65 years. Among those aged 0–4 years, boys were more likely than girls to have a visit with asthma recorded, but for the older age groups, 18–64 and ≥65, women with asthma documented were more likely than men to have an ED visit. The difference by sex for those aged 5–17 years was not statistically significant.

**Source: **NCHS, National Hospital Ambulatory Medical Care Survey, 2014–2015.

